# Efficacy of selective laser trabeculoplasty on lowering intraocular pressure fluctuations and nocturnal peak intraocular pressure in treated primary open-angle glaucoma patients

**DOI:** 10.1007/s00417-022-05897-y

**Published:** 2022-11-23

**Authors:** Karin R. Pillunat, Greta A. Kocket, Robert Herber, Carolin S. Jasper, Janine Lenk, Lutz E. Pillunat

**Affiliations:** Department of Ophthalmology, Medical Faculty Carl Gustav Carus, Technical University, Fetscherstrasse 74, 01307 Dresden, Germany

**Keywords:** Selective laser trabeculoplasty, Circadian tension curves, 24-h IOP fluctuation, Nocturnal peak IOP

## Abstract

**Purpose:**

To investigate the efficacy of adjunctive selective laser trabeculoplasty (SLT) in reducing 24-h intraocular pressure (IOP) fluctuations and nocturnal IOP peaks.

**Methods:**

In this prospective interventional case series, 157 medically treated eyes of 157 patients with primary open-angle glaucoma (POAG) who were assigned SLT to further reduce IOP were consecutively included. Each patient had a complete glaucoma work-up and 24-h IOP monitoring (6 measurements, including one in the supine position) taken before and on average 6 months after SLT. The main outcome measures were the reduction of 24-h IOP fluctuations and nocturnal peak IOP. Secondary outcome measures were success rates, factors influencing the reduction of high 24-h IOP fluctuations and nocturnal peak IOP, complications, and severe adverse events.

**Results:**

Medicated mean 24-h IOP (mmHg) was statistically significantly reduced from 15.1 ± 2.6 to 13.8 ± 2.4 (*P* < 0.001) and IOP fluctuations from 6.5 ± 2.7 to 5.4 ± 2.6 (*P* < 0.001) 6 months after SLT. Ninety-four eyes (59.9%) initially had high IOP fluctuations (more than 5 mmHg). These were reduced from 8.1 ± 2.3 to 5.6 ± 2.7 at 6 months (*P* < 0.001). Fifty-two eyes (55.3%) had fluctuations below 5 mmHg post-SLT which was defined as success. Fifty-one patients (32.5%) had nocturnal IOP peaks. In these cases, nocturnal IOP was reduced by 19.2% from 20.1 ± 3.4 to 16.2 ± 3.3 mmHg at 6 months (*P* = 0.001).

**Conclusions:**

The current study demonstrates that adjunctive SLT not only reduces mean 24-h IOP in treated POAG patients, but also has an additional benefit in reducing IOP fluctuations and nocturnal peak IOP.

**Trial registration:**

Clinical trial registration: NCT02959242.


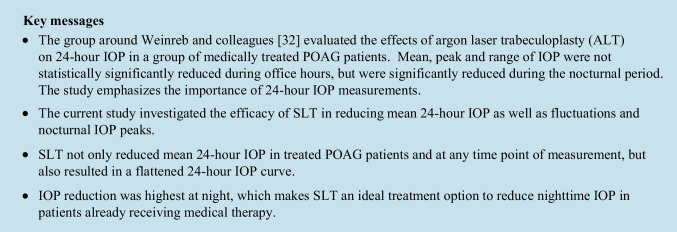


## Introduction

Although glaucoma is a multifactorial disease, elevated intraocular pressure (IOP) remains the most important risk factor and the reduction of IOP is currently still the only evidence-based therapy to treat patients with any form of glaucoma [1–3]. The main treatment goal is to maintain visual function by preventing or slowing the rate of glaucomatous optic nerve damage. If maximum-tolerated topical medication fails to control the disease, laser trabeculoplasty such as selective laser trabeculoplasty (SLT) is often applied before more invasive surgical procedures are carried out. SLT is effective and safe as primary [4, 5] as well as adjunctive therapy [6, 7]. The complete mode of action is still not completely understood; however, in many cases, the transmission of electromagnetic energy to the trabecular meshwork results in an improved trabecular aqueous outflow and subsequently, in a decrease of IOP [8–10].

Usually, IOP is monitored with single measurements during office hours. However, about two-thirds of patients show higher IOPs outside of this timeframe [11, 12] with peak IOPs mostly appearing at night in the supine position [13–15], possibly reflecting a rise in episcleral venous pressure. Since IOP is central for glaucoma management, the verification of IOP at baseline and the evaluation of treatment efficacy of any IOP-lowering therapy should not only target daytime IOP, but also nocturnal IOP, peak IOP, and IOP fluctuations [16].

In a companion paper, the ability of adjunctive SLT to further lower IOP in treated primary open-angle glaucoma (POAG) patients was investigated and predictors for IOP lowering success were identified [6]. The present study aims to analyze 24-h IOP before and 6 months after SLT, which allows for the assessment of changes in IOP at different points in time during the 24 h, as well as IOP fluctuations and nocturnal peak IOP. IOP-lowering medication was not changed during follow-up, thereby allowing for an appraisal of the sole effect of SLT.

## Materials and methods

In this prospective interventional case series, all patients with POAG, who were scheduled for SLT because insufficient control over the disease was achieved by maximum-tolerated topical IOP-lowering medication, were consecutively enrolled. SLT was used as an add-on therapy and to delay surgery. In the case of both eyes being eligible, one eye was randomly selected. The decision to perform SLT was made after baseline 24-h IOP measurements if the individual target IOP was not reached or the visual field progressed and if it was the preference of the patient. Exclusion criteria were ophthalmic surgery other than uncomplicated phacoemulsification at least 3 months prior to study inclusion and patients under the legal age, as well as open-angle glaucoma with pseudoexfoliation or pigment dispersion syndrome.

The study protocol was approved by the Institutional Review Board of the Medical Faculty Carl Gustav Carus of the Technische Universität Dresden, Germany, and followed the tenets of the Declaration of Helsinki. All participants signed a written informed consent. Clinical trial registration: NCT02959242.

POAG included high (HPG) and normal pressure glaucoma (NPG) patients. HPG was defined as POAG with a history of repeatedly elevated untreated IOPs higher than 21 mm Hg in the absence of other secondary causes for IOP elevation, open drainage angles on gonioscopy, a glaucomatous optic disc with characteristic glaucomatous cupping, focal or diffuse thinning of the neuroretinal rim, nerve fiber layer defects and/or corresponding visual field defects, but no other ocular or systemic diseases that might cause visual field defects. NPG was defined as POAG with a history of repeatedly normal untreated IOPs equal or less than 21 mm Hg; otherwise, the same definitions applied as for HPG. NPG patients more often suffered from vasodysregulative or vasosclerotic dysfunction, which was assessed with vascular-related questions [17].

Baseline recordings included sex, age, number, and classes of IOP lowering medications as well as previous surgeries. A thorough ophthalmic examination included refraction, best spectacle-corrected visual acuity (BCVA), slit-lamp biomicroscopy of the anterior segment, 24-h IOP measurements taken with Goldmann applanation tonometry (GAT), gonioscopy, and dilated fundus examination with a 90-diopter lens. A complete glaucoma work-up included automated perimetry (Swedish interactive threshold algorithm standard 30–2 program; Carl Zeiss Meditec, Dublin, CA, USA), confocal scanning laser ophthalmoscopy (HRT II, Heidelberg Engineering Inc., Heidelberg, Germany), and scanning laser polarimetry (Nerve Fibre Analyzer GDxPRO, Carl Zeiss Meditec, Dublin, CA, USA).

Measurements of 24-h IOP were taken during an overnight hospital admission at 1, 4, 7 and 10 pm in a sitting position at the slitlamp with a calibrated Goldmann tonometer (Haag-Streit, Koeniz, Switzerland), at midnight in a supine position using the handheld calibrated Perkins MK3 tonometer (HS Clement Clark Ophthalmic, Haag-Streit UK) and at 7 am again in a sitting position at the slitlamp before application of IOP-lowering medications. These are the specified IOP monitoring times for all glaucoma patients during a one-day glaucoma work-up. Masked observers took one measurement within half an hour at each point in time, as it is practice in daily clinical routine [18]. A previous study by Wozniak et al. [13], which was performed in the same setting, analyzed repeated IOP measurements in the supine position during the night and found, that the midnight measurement was on average the highest. In order not to disturb the patients sleep too much, the midnight time point was chosen as representative for nocturnal IOP in the supine position.

Large 24-h IOP fluctuations were defined as the difference between the highest and the lowest IOP measurement being more than 5 mmHg. Nocturnal IOP peaks were defined as the IOP taken at midnight in the supine position being more than 3 mmHg above the mean of the other 5 measurements.

SLT was performed by a single experienced surgeon (KRP) with the Ellex SLT laser (SoloTM, ellex Inc., Adelaide, Australia) using a Latina SLT goniolens (Ocular Instr., Bellevue, USA) to visualize the trabecular meshwork [6]. The initial energy level was set to 0.8 mJ and adapted according to the grade of angle pigmentation and bubble formation. Since the cohort was quite homogenous with 100% Caucasians and the inclusion of only open-angle glaucoma patients, the trabecular meshwork was very little pigmented, mostly grade 1 and rarely grade 2 out of 4. One hundred non-overlapping spots were applied in a single session to 360° of the trabecular meshwork. No anti-inflammatory medications were applied postoperatively. The number of glaucoma medications and substances was not changed or discontinued during follow-up to allow an evaluation of the sole effect of SLT.

Postoperative examinations at the 6-month follow-up included refraction, BCVA, slit-lamp examination of the anterior and posterior segment, GAT (average of 6 measurements at 1, 4, 7, and 10 p.m., at midnight in a supine position, and at 7 a.m. as described above) as well as a full glaucoma work-up as previously described.

Primary endpoints were the reduction of medicated mdIOP (mean of 6 measurements including a measurement at midnight in a supine position) and at the individual times of measurement, of high diurnal IOP fluctuations, and nocturnal peak IOP. Secondary outcomes were SLT success rates, factors influencing IOP reduction, postoperative complications, and severe adverse events. Success was defined as post-SLT IOP fluctuations of less than 5 mmHg and nocturnal supine IOP being less than 3 mmHg above the other 5 measurements in a sitting position.

### Statistical analysis

Based on the knowledge from the EMGT study [19] that any mmHg IOP reduction slows progression, a sample size of at least 75 patients (alpha = 0.05; power = 0.80) was required (G Power 3.1.9.2. sample size software; University of Duesseldorf, Germany).

Statistical analysis was performed using SPSS (version 25, IBM Statistics; New York, USA). Normal distribution was tested with the Kolmogorow-Smirnow-test. Normally distributed data were expressed as mean and standard deviation and non-normally distributed data as median and interquartile range (IQR). Paired *t*-test was used to analyze preoperative and postoperative data. Chi^2^ test was used to analyze dichotomous data. To examine the correlation, Pearson correlation test was used. A *P*-value lower than 0.05 was considered as statistically significant.

## Results

**Fig. 1:**
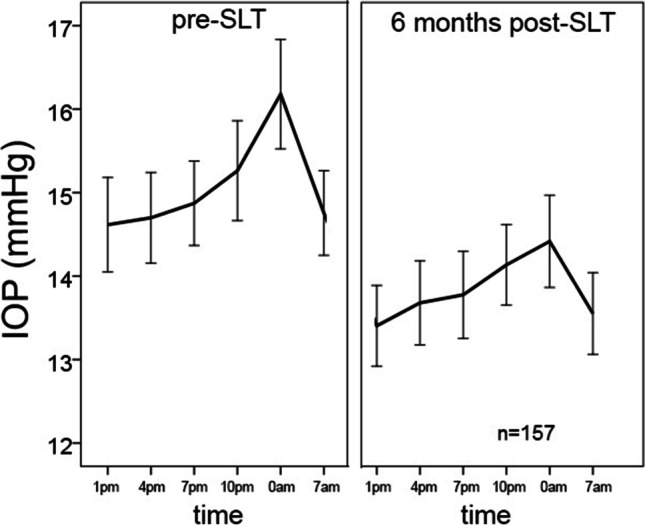
IOP was statistically significantly reduced at all six time points of measurement with maximum reduction at night (*P* < 0.001), the tension curve pattern remained similar post-SLT. Mean and standard deviation

Demographics and ocular parameters are shown in Table 1. Medicated mean diurnal IOP was statistically significantly reduced from 15.1 ± 2.6 to 13.8 ± 2.4 mmHg (*P* < 0.001), 6 months after SLT. IOP was statistically significantly reduced at all six time points of measurement (*P* < 0.001; Table 2; Fig. 1) with maximum reduction at night.

**Table 1:** Demographic data and ocular parameters. Absolute and relative numbers, means and standard deviation, and median with interquartile range

No. of eyes/patients	157/157
Eyes (right/left¸ No./%)	78/79 (50%/50%)
Age (years)	68.2 ± 11.1
Gender (female/male; No./%)	88/69 (56%/44%)
BCVA (logMAR)	0.185 ± 0.245
Pseudophacic (No./%)	50 (31.8%)
CCT in µm	539.4 ± 34.8
HPG/NPG (No./%)	115/42 (73.2%/26.8%)
MD in dB	-7.5 (IQR: -15.0…-4.0)
MD > − 6 dB	63 (40%)
− 12 dB < MD < − 6 dB	37 (24%)
MD < − 12 dB	57 (36%)
PSD in dB	6.8 (IQR: 2.6…10.7)
Preoperative mdIOP (mmHg)	15.1 ± 2.6
Preoperative diurnal fluctuations > 5 mmHg (No.)	94 (59,9%)
Preoperative nocturnal peak IOP > 3 mmHg (No.)	51 (32.5%)
IOP at time of diagnosis (mmHg)	25.7 ± 7.1
Known duration of disease (years)	11.8 ± 8.1
Number of medications	2.9 ± 0.9
Substances (No. / %)	PGA: 143 (91.1%) β-Bl: 121 (77.1%) α-Ag: 59 (37.6%) CAI: 100 (63.7%) pilocarpin: 27 (17.2%)
SLT parameters:	
Spots (No.) Laser Energy (mJ)	97 ± 9 0.8 ± 0.1

*No*., number; *BCVA*, best corrected visual acuity; *CCT*, central corneal thickness; *HPG*, high pressure glaucoma; *NPG*, normal pressure glaucoma; *MD*, mean deviation; *PSD*, pattern standard deviation; *IQR*, interquartile range; *mdIOP*, mean diurnal intraocular pressure; *PGA*, prostaglandin analogues; *ß-Blocker*, Betablockers; *α-Ag*, Alphaagonists; *CAI*, carboanhydrase inhibitors; *mJ*, millijoule

**Table 2:** Reduction of mean IOP at the different time points. IOP measurements were taken at 1, 4, 7, and 10 pm in a sitting position, at midnight in a supine position and at 7 am again in a sitting position with Goldmann applanation tonometry. Means and standard deviation

Time	Preoperative (mmHg)	Postoperative (mmHg)	Mean change (mmHg)	Mean change (%)	*P*-value
1 pm	14.6 ± 3.6	13.4 ± 3.1	− 1.2 ± 3.3	− 8.2	< 0.001
4 pm	14.1 ± 3.4	13.7 ± 3.2	− 1.0 ± 3.2	− 7.1	< 0.001
7 pm	14.9 ± 3.2	13.8 ± 3.3	− 1.1 ± 3.2	− 7.4	< 0.001
10 pm	15.3 ± 3.8	14.1 ± 3.0	− 1.1 ± 3.1	− 7.2	< 0.001
0 am	16.2 ± 4.1	14.4 ± 3.5	− 1.8 ± 3.6	− 11.1	< 0.001
7 am	14.8 ± 3.2	13.6 ± 3.1	− 1.2 ± 3.1	− 8.1	< 0.001

Overall, IOP fluctuations were statistically significantly reduced (Table 3) from 6.5 ± 2.7 to 5.4 ± 2.6 mmHg (*P* < 0.001). Ninety-four eyes (59.9%) initially had large IOP fluctuations. These were reduced from 8.1 ± 2.3 to 5.6 ± 2.7 mmHg at 6 months (*P* < 0.001). Out of these, 52 eyes (55.3%) had diurnal IOP fluctuations below 5 mmHg post-SLT which was defined as success. In these cases, IOP fluctuations were reduced from 7.6 ± 2.3 to 3.7 ± 1.2 at 6 months (*P* < 0.001). Nocturnal IOP peaks (Table 3) were seen in 51 patients (32.5%) pre-SLT. Nocturnal IOP in these cases was reduced by 19.2% from 20.1 ± 3.4 to 16.2 ± 3.3 mmHg at 6 months (*P* = 0.001). The difference between IOP measured at midnight in the supine position and the mean of the other 5 measurements was reduced from 5.4 ± 2.1 to 2.6 ± 3.5 (*P* = 0.001). In 42 eyes (82.4%), nocturnal peaks were decreased. A reduction to below 3 mmHg difference in comparison to the 5 measurements taken in a sitting position was seen in 27 eyes (52.9%), which was again defined as success. In these cases, nocturnal IOP was reduced by 28.5% from 20.6 ± 3.2 to 14.7 ± 2.9 (*P* = 0.001), which is a reduction of the difference from 5.2 ± 2.2 to 0.1 ± 2.2 mmHg at 6 months.

**Table 3:** Pre- and postoperative IOP-values. Means and standard deviation

*N* = 157	Preoperative	Postoperative	*P* value
mdIOP (mmHg)	15.1 ± 2.6	13.8 ± 2.4	< 0.001
IOP fluctuations (mmHg)	6.5 ± 2.7	5.4 ± 2.6	< 0.001
Preoperative diurnal fluctuations > 5 mmHg (*N* = 94)	8.1 ± 2.3	5.6 ± 2.7	< 0.001
Postoperative diurnal fluctuations < 5 mmHg (*N* = 52)	7.6 ± 2.3	3.7 ± 1.2	< 0.001
Preoperative nocturnal peak IOP > 3 mmHg (*N* = 51)	20.1 ± 3.4	16.2 ± 3.3	0.001
Postoperative nocturnal peak IOP < 3 mmHg (*N* = 27)	20.6 ± 3.2	14.7 ± 2.9	0.001

**Fig. 2:**
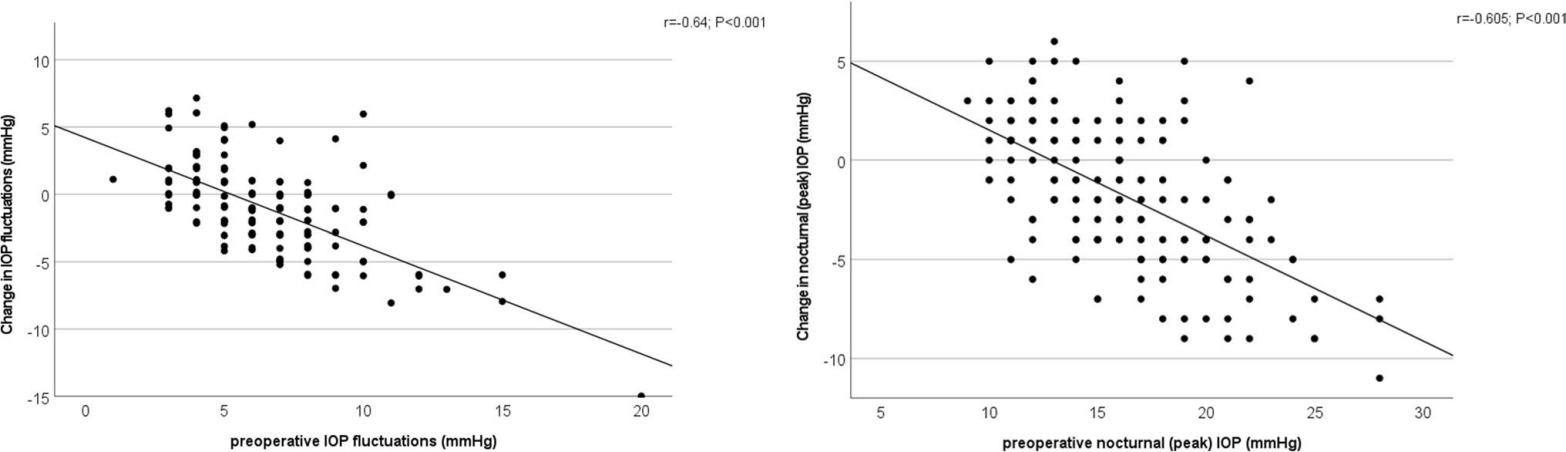
Correlation between pre- and post-SLT IOP fluctuations (**a**) and nocturnal peak IOP (**b**). A higher range of fluctuation and a higher pre-SLT nocturnal peak IOP were the only predictors of the efficacy of reducing these fluctuations (r =  − 0.64; *P* < 0.001) or the nocturnal IOP peaks (r =  − 0.605; *P* < 0.001). Pearson correlation

A higher pre-SLT IOP was the only factor predicting SLT efficacy [6]. A higher range of fluctuation was the only predictor of the efficacy of reducing these fluctuations (r =  − 0.64; *P* < 0.001; Fig. 2a). If IOP fluctuation was higher than 5 mmHg pre-SLT (94 eyes), 76.6% (72 eyes) showed reduced fluctuations 6 months post-SLT. The same was true for nocturnal IOP peaks, the higher the pre-SLT nocturnal IOP peak, the better was the reduction post-SLT (r =  − 0.605; *P* < 0.001; Fig. 2b). Age, sex, whether treated HPG or NPG, stage of the disease (MD, PSD), pachymetry, pseudophacic status, type, and number of IOP-lowering substances had no influence on post-SLT success in reducing IOP fluctuations or nocturnal IOP peaks. We did not see any postoperative complications, clinically significant inflammatory reactions or severe adverse events.

## Discussion

Although medical hypotensive therapy is effective in lowering IOP, maximum-tolerated topical medication sometimes still fails to control the disease. Besides not reaching the individual target IOP, 24-h IOP fluctuations and nocturnal IOP peaks are often not flattened with medical therapy, even in patients treated with prostaglandin analogues and carbonic anhydrase inhibitors, which are known to lower both diurnal and nocturnal IOP.

This study investigated the effectiveness of SLT adjunctive to maximum-tolerated topical medical therapy to further reduce 24-h IOP in eyes diagnosed with POAG, as well as IOP fluctuations and nocturnal IOP peaks. Adjunctive SLT not only reduced mean 24-h IOP in treated POAG patients, but also had an additional benefit in reducing IOP fluctuations and nocturnal peak IOP in about 50% of the cases with wide fluctuations and where IOP showed to be highest at night.

Larger short-term IOP fluctuations can be a risk factor for progression in open-angle [20, 21] as well as angle-closure glaucoma [22]. Most studies on progression and IOP fluctuations do not include nocturnal IOP measurements or measurements in the supine position. This is one reason why evidence in literature is heterogeneous [23]. The mechanisms of IOP fluctuations on glaucoma progression are not fully understood yet. The initial site of glaucomatous injury to axons and extracellular matrix is the area of the lamina cribrosa [24]. IOP-related stress and strain to connective tissues at the optic nerve head play an important role in the pathophysiology of glaucomatous damage [25]. In addition, IOP fluctuations could cause unstable blood flow and nutrient supply as a consequence from the impaired autoregulation associated with glaucoma [26]. Therefore, smoothing out IOP fluctuations should be one of the treatment goals in glaucoma patients. Prostaglandin analogues have been proven to reduce IOP during both day and night as well as 24-h IOP fluctuations effectively [27, 28]. Compared to SLT, latanoprost [29] better controlled IOP fluctuations in newly diagnosed OHT (ocular hypertension) or POAG patients. In the current study, SLT was used as additional IOP-lowering therapy to already maximally treated glaucoma patients, of which 91.1% used prostaglandin analogues and 63.7% carbonic anhydrase inhibitors; both antiglaucoma therapies are known to lower diurnal and nocturnal IOP. Nevertheless, 59.9% (94 eyes) showed 24-h IOP fluctuations of more than 5 mmHg, which could be flattened in more than half of the cases (55.3%, 52 eyes) after SLT, and 32.5% (51 eyes) had nocturnal IOP peaks 3 mmHg higher than the 5 daytime measurements, which also could be lowered in about half of the cases (52.9%, 27 eyes). Possibly, adherence to medication plays an additional role.

IOP and aqueous humor flow have a circadian rhythm with peak IOP frequently occurring at night in the supine sleeping position [13–15]. This can partly be explained by an increase in episcleral venous pressure in the supine position. In the current study, SLT statistically significantly lowered IOP at each single time point of measurement with the most significant reduction observed at nighttime (Fig. 1; Table 2). Interestingly, the tension curve pattern remained similar in shape after SLT-treatment (Fig. 1).

Nocturnal IOP might be more critical than daytime IOP as a lower systemic blood pressure can occur simultaneously with high IOPs at night. Together with an impaired autoregulation [26], this might reduce ocular blood flow and nutrient supply below a critical level, thereby resulting in optic nerve damage [30]. Glaucoma treatment should ideally maximally reduce IOP over 24 h and minimally influence blood pressure. In a previous study, we demonstrated that SLT probably does not induce any pharmacological changes effecting systemic blood pressure or ocular blood flow as topical IOP-lowering medication might do [31]. Therefore, SLT is an ideal option to reduce nighttime IOP, as has already been shown in a study by Lee et al. [32] using ALT.

SLT is not effective in all POAG eyes, however. Out of 157 medically treated eyes, 121 (77.1%) were responders and showed a reduction of mean diurnal IOP after SLT [6]. Fifty-five percent of patients with large IOP fluctuations and 52.9% with nocturnal peak-IOP showed successfully reduced IOP values, post-SLT. The reason why a considerable proportion of eyes do not adequately respond to SLT is still not known. POAG probably affects not only the trabecular meshwork (TM) but post-TM structures like Schlemm´s canal and the collector channels as well, with a genetic variability possibly playing a role [33]. If a proportion of high IOP is rather due to Schlemm´s canal or collector channel pathology, a TM-targeted therapy, such as SLT, is not effective [34]. Consequently, non-responders with advanced disease should probably be advised to undergo drainage surgery or cyclodestructive procedures.

One of the limitations of the current study is that 24-h tension curves were taken using IOP measurements at intervals a few hours apart and not continuously as is possible with a contact lens sensor (CLS), for example. However, the recordings with such a CLS can only measure IOP-related fluctuations in arbitrary units, plotting a graph of the 24-h IOP rhythm. These arbitrary units cannot easily be converted into the gold standard unit of IOP measurement, which is mmHg. However, the 7 hours between 0 and 7 am and the 6 hours between 7 am and 1 pm could be time intervals too long to exclude additional IOP peaks. Besides, IOP measurements were taken only once at every specific IOP monitoring time. Furthermore, it was designed as a single-arm study without a placebo group, since this would be ethically not acceptable in such an advanced glaucoma cohort. Also, the influence that a higher or lower central corneal thickness might have on discrimination between HPG and NPG was not accounted for. In addition, the study only included Caucasian patients with medically treated POAG. Our findings are not generalizable to other ethnicities, other subtypes of glaucoma, or untreated patients. Lastly, follow-up time was rather short. Strengths of the study are the standardized 24-h IOP measurements taken always at the same time of the day, including a measurement at night in the supine position, rather than IOPs taken at non-standardized points in time. Further, the inclusion of treated HPG and NPG patients with baseline IOP being rather low already. In addition, the individual IOP-lowering medication was not changed throughout the study period.

In conclusion, adjunctive SLT not only reduced mean 24-h IOP in treated POAG patients, but also had an additional benefit in reducing IOP fluctuations and nocturnal peak IOP. This resulted in a flattened 24-h IOP curve. IOP reduction was highest at night, which makes SLT an ideal treatment option to reduce nighttime IOP in patients already receiving medical therapy, even more so as it does not influence systemic blood pressure [31]. Since SLT is safe, easy to perform, and independent of patient compliance, it can definitely be considered as a good treatment option in treated POAG eyes not at target as any mmHg IOP reduction slows progression [19].
